# Alteration of ACTH and Cortisol Levels After Estradiol Valerate Treatment in a Male Subject With Gender Dysphoria: A Case Report

**DOI:** 10.3389/fendo.2019.00751

**Published:** 2019-11-05

**Authors:** Takatoshi Anno, Fumiko Kawasaki, Ryo Shigemoto, Shintaro Irie, Tomoatsu Mune, Kohei Kaku, Hideaki Kaneto

**Affiliations:** ^1^Department of General Internal Medicine 1, Kawasaki Medical School, Okayama, Japan; ^2^Department of Diabetes, Metabolism and Endocrinology, Kawasaki Medical School, Kurashiki, Japan

**Keywords:** estrogen replacement therapy, male-to-female transsexual subjects, ACTH, cortisol, estradiol levels

## Abstract

**Background:** The number of subjects with gender dysphoria has been increasing. In general, male-to-female transsexual subjects are treated with estradiol valerate therapy. In this report, we showed the time course of ACTH and cortisol levels after estradiol valerate injection in a male subject with gender dysphoria. It seemed that alteration of estradiol levels influenced ACTH and cortisol levels via some pathway.

**Case presentation:** A 31-year-old man with estradiol valerate therapy for gender dysphoria was referred due to an elevation of serum cortisol levels. She started hormone therapy at 26 years old. Her laboratory analyses showed an elevation of plasma ACTH and cortisol levels. There were no remarkable changes in the adrenal gland and pituitary gland. Her estradiol levels were elevated 7 days after estradiol valerate injection, but they were not detected 18 days after such treatment. Interestingly, plasma ACTH and serum cortisol levels were moderately decreased 7 days after estradiol valerate injection, but both were markedly elevated 18 days after such treatment.

**Conclusions:** We should bear in mind the possibility of elevation in plasma ACTH and serum cortisol levels when we start estradiol valerate injection in subjects with gender dysphoria. In addition, we may need to check ACTH and cortisol levels when we use estrogen replacement therapy for a long period of time in subjects with gender dysphoria.

## Background

The number of subjects with gender dysphoria has been increasing in recent years, and some of them have been treated with hormone replacement treatment and/or surgery. Accordingly, clinical practice guidelines were provided for hormone therapy in subjects with gender dysphoria or transsexualism (1). In general, male-to-female transsexual subjects are treated with estrogens, anti-androgens, and a GnRH agonist for a long period of time. However, few studies have compared hormone levels between before and after long-term estrogen replacement therapy for gender dysphoria (2). Most other studies have not even measured cortisol levels after estrogen replacement therapy and thereby it remains unknown whether various hormone levels are actually altered or not after such hormone replacement therapy.

In general, it is known that cortisol levels increase with age in women. In addition, cortisol levels in premenopausal woman are lower compared to those in men at the same age (3). After menopausal transition, cortisol levels are increased in women (4). Previously, several studies have shown that oral estrogen replacement therapy increased total cortisol levels in women (5, 6), although some reports did not show such differences (7). It was reported that after male-to-female transsexual reassignment surgery, cortisol levels were not markedly altered, although androgen levels were reduced (8). In other words, it seemed that reduced androgen levels did not change cortisol levels in subjects with male-to-female transsexual reassignment surgery.

In this report, however, we show a case of periodically elevated ACTH and cortisol levels related to estradiol valerate injection in a male subject with gender dysphoria.

## Case Presentation

A 31-year-old man with a 5-year history of hormone replacement treatment for gender dysphoria was referred to Kawasaki Medical School due to the elevation of serum cortisol levels. She started hormone therapy at 26 years old with oral drugs (conjugated estradiol or estrogens), although she did not undergo male-to-female transgender surgery. Recently, she has repeatedly received 10 mg estradiol valerate therapy every 3 weeks. Upon physical examination, she exhibited no remarkable change, which is often observed in Cushing's syndrome, and she had no subjective symptoms related to such a disease. Her height and body weight were 170.0 cm and 59.0 kg. Her vital signs were: heart rate 80 beats/min; blood pressure 129/70 mmHg; body temperature 37.2°C. Laboratory data were as follows: white blood cell count, 5,130 μL (neutrophil 46.6%, eosinophil 2.1%, and lymphocyte 42.5%); red blood cell count, 409 × 10^4^/μL; hemoglobin, 12.9 g/dL; platelet, 19.9 × 10^4^/μL; Na, 138 mmol/L; K, 3.9 mmol/L. Renal and liver function was within normal range. Laboratory analyses (11 days after estradiol valerate treatment) showed that this patient had an elevation of plasma ACTH, serum cortisol (ACTH, 139.6 pg/mL; cortisol, 38.8 μg/dL; DHEA-S, 135 μg/dL), and hyperprolactinemia (PRL, 49.8 ng/mL) as well as elevated estradiol levels (E2, 99 pg/mL) and hypogonadism (LH, 1.16 mIU/mL; FSH, 0.96 mIU/mL; testosterone, 0.8 pg/mL). We think that most of the changes were presumably due to the effect of estradiol valerate treatment as this examination was performed 11 days after the estradiol valerate injection and the estradiol level was actually elevated. Although we failed to compare ACTH and/or cortisol levels between before and after estradiol valerate treatment, ACTH and cortisol levels after the treatment were markedly higher compared to the reference range. There were no remarkable changes in the adrenal gland upon abdominal computed tomography and pituitary gland upon pituitary magnetic resonance imaging. ACTH, cortisol, PRL, and E2 were measured with electro chemiluminescence immunoassay (ECLIA) (SRL, Inc., Tokyo, Japan). DHEA-S was measured with chemiluminescent enzyme immunoassay (CLEIA) (SRL, Inc., Tokyo, Japan). LH and FSH were measured with chemiluminescent Immunoassay (CLIA) (SRL, Inc., Tokyo, Japan).

We examined the hormone level profile under resting and fasting conditions during two course treatments with estradiol valerate injection. She visited our office at 8 am under fasting conditions and we drew blood sampling for a blood test after resting for 30 min on the bed. In course 1, estradiol level was detected 14 days after the injection (11 pg/mL) but was not detected 21 days later. Since estradiol level in this subject was not detected before the treatment in course 2 (Table 1), it seemed that the detected estradiol level (11 pg/mL) was influenced by estradiol valerate injection. ACTH and cortisol levels tended to be higher 21 days after the treatment (51.5 pg/mL and 32.4 μg/dL) compared to 14 days (45.0 pg/mL and 31.6 μg/dL). Thus, it seemed that ACTH and cortisol levels were influenced by estradiol valerate treatment and that there might be some time lag between the increase of estradiol level and the subsequent increase of ACTH and cortisol levels. In course 1, however, we failed to examine estradiol, ACTH, and cortisol levels before the treatment, and the change of ACTH and cortisol levels was not large enough.

**Table 1:** Hormone level profile before and after estradiol valerate injection.

**Serum hormone level**	**Before estradiol** ** valerate injection**	**7 days after** ** the injection**	**18 days after** ** the injection**
ACTH (pg/mL)	51.5	28.3	85.4
Cortisol (μg/dL)	32.4	28.3	38.9
LH (mIU/mL)	9.83	7.26	5.90
FSH (mIU/mL)	8.48	2.31	4.67
Estradiol (E2) (pg/mL)	0	276	0
Progesterone (ng/mL)	0.31	0.24	0.39
Testosterone (pg/mL)	5.7	2.9	3.2
Prolactin (ng/mL)	21.02	21.78	40.39

**Figure 1:**
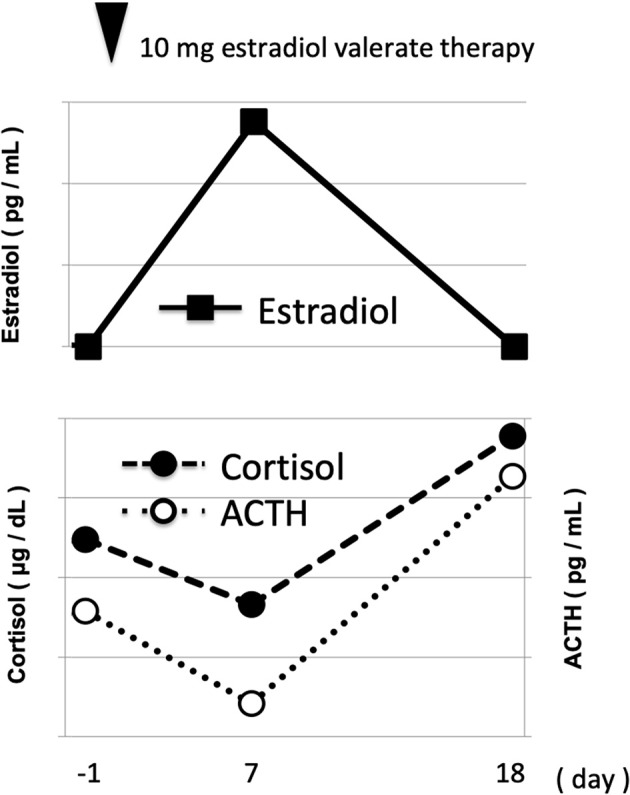
Time course of hormone level profile under resting and fasting conditions before and after 10 mg of estradiol valerate injection. White circle, estradiol; pink circle, cortisol; blue circle, ACTH.

In order to examine the possible association of hormone replacement therapy with elevated ACTH and cortisol levels, therefore, we next precisely measured ACTH and cortisol levels under resting and fasting conditions before and after estradiol valerate treatment. As shown in Table 1 and Figure 1, estradiol level in this subject was not detected at all before estradiol valerate injection (0 pg/mL), but it was markedly elevated 7 days after the injection (276 pg/mL) and was not detected again at all 18 days later (0 pg/mL). ACTH and cortisol levels in this subject were 51.5 pg/mL and 32.4 μg/dL, respectively, before estradiol valerate injection. ACTH and cortisol levels were moderately decreased 7 days after estradiol valerate injection (28.3 pg/mL and 28.3 μg/dL, respectively), and, interestingly, both of them were markedly elevated 18 days after such treatment (ACTH, 84.5 pg/mL; cortisol, 38.9 μg/dL) (Figure 1). In addition, as shown in Table 1, the prolactin level was also increased 18 days after estradiol valerate treatment (21.02–40.39 ng/mL), suggesting the possibility that estrogen therapy influences pituitary gland function. In order to examine the possible cause of elevated ACTH and cortisol levels in more detail, we tried to perform various tests, including the Dexamethasone load test and corticotropin-releasing hormone tolerance test, but this subject hesitated to continue such hormone therapy with estradiol valerate because of the appearance of a symptom similar to menopause. In addition, there were no remarkable changes in the adrenal gland upon abdominal computed tomography and pituitary gland upon pituitary magnetic resonance imaging. Therefore, we observed her without additional tests in accordance with her wishes.

## Discussion and Conclusions

In this report, we showed the time course of ACTH and cortisol levels after estradiol valerate injection in a male subject with gender dysphoria. As shown in Figure 1, it seemed that alteration of estradiol levels influenced ACTH and cortisol levels via some pathway. Finally, ACTH and cortisol levels were markedly increased after estradiol valerate treatment. To the best of our knowledge, this is the first report showing the time course of ACTH and cortisol levels after estrogen replacement therapy in a subject with gender dysphoria. Interestingly, there was some time lag between the increase of estradiol level and the subsequent increase of ACTH and cortisol levels. Although its precise mechanism remains unknown, we assume that such a time lag is, at least in part, induced by the metabolic rate of estradiol. It is possible that a metabolite of estradiol influenced ACTH and cortisol levels. In addition, since estrogen is originally a female hormone, a high concentration of estrogen might exert some undesirable effects in male subjects compared to female ones.

There are several possible mechanisms that can explain how ACTH and cortisol levels were increased after estradiol valerate treatment. One possibility is that estrogen therapy directly or indirectly influenced ACTH secretion from the pituitary gland, which facilitated cortisol secretion from the adrenal gland. It is known that in pregnant women the hypothalamic-pituitary-adrenal (HPA) axis is activated and hypercortisolism is observed (9, 10). In situations of pregnancy, therefore, elevated estradiol levels might be, at least in part, associated with HPA axis activity and elevated cortisol levels. Moreover, in general, estradiol levels are higher in the luteal phases rather than in the follicular phases, and cortisol levels are higher in the follicular phases rather than in the luteal phases (11, 12). Therefore, it is possible that there is some time lag between the increase of estradiol level and the subsequent increase of ACTH and cortisol levels. Furthermore, HPA axis activity might have something to do with sex-specific effects. The HPA axis is influenced by various body stress responses, and it is noted that the sex difference is observed in this HPA axis system (13). The HPA axis in females initiates more rapidly and produces a greater output of stress hormones, and estrogens decrease HPA activity in adult women. HPA axis activity through the brain and peripheral signals is sex-specific in many cases. It is likely that ovarian steroids have some effects on HPA function as ovarian steroid receptors are widely expressed in female brain regions that regulate both direct and indirect activation and negative feedback of the HPA axis (14).

Indeed, in this case, the prolactin level was increased by estrogen therapy, suggesting the possible effect of estrogen on the pituitary gland. However, since it is known that the prolactin level was increased by various kind of medications, we cannot deny the possibility that the increase of the prolactin level in this subject was simply a side effect of the estradiol valerate. Therefore, it would be necessary to perform some other experiments in order to demonstrate how ACTH and cortisol levels are augmented by estrogen therapy. Another possibility is that elevated estradiol suppressed transcription of 11-β hydroxysteroid dehydrogenase (11-β HSD), which is known to regulate intracellular cortisol production and affect the local organization, such as the liver and adipose tissue. It is possible that, if local cortisol levels are decreased in the pituitary and/or hypothalamus via the above pathway, ACTH levels are increased by decreased cortisol level (15). However, there is evidence that estrogen influences 11B-HSD (16, 17), and it remains unclear how ACTH and cortisol levels were increased after estradiol valerate treatment.

There is a limitation in this case report. This subject had symptoms similar to menopause with interruption of therapy and thus hesitated to continue such hormone therapy with estradiol valerate. We therefore failed to perform a Dexamethasone load test and to measure free cortisol and CBG levels, and we measured DHEA-S levels only once. In addition, since she had no symptoms related to Cushing's syndrome, it is possible that elevated ACTH and cortisol were biologically inactive.

Taken together, we should bear in mind the possibility of elevation in plasma ACTH and serum cortisol levels when we start estradiol valerate injection in subjects with gender dysphoria. Moreover, although long-term therapy with estradiol valerate is necessary for gender dysphoria, long-term side effects of estrogen therapy remain unknown. Therefore, it would be safer to check ACTH and cortisol levels regularly, even in subjects without symptoms related to Cushing's syndrome, especially when we use estrogen replacement therapy for a long period of time in subjects with gender dysphoria. It is needless to say that when subjects receiving estrogen therapy have symptoms related to Cushing's syndrome we should check ACTH and cortisol levels immediately.

## Ethics Statement

Written informed consent was obtained from the individual(s) for the publication of any potentially identifiable images or data included in this article.

## Author Contributions

TA and HK researched data and wrote the manuscript. FK, RS, SI, TM, and KK researched data and contributed to the discussion.

### Conflict of Interest

The authors declare that the research was conducted in the absence of any commercial or financial relationships that could be construed as a potential conflict of interest.

## References

[1] Hembree WC Cohen-Kettenis P Delemarre-van de Waal HA Gooren LJ Meyer WJ III Spack NP. Endocrine treatment of transsexual persons: an Endocrine Society clinical practice guideline. J Clin Endocrinol Metab. (2009) 94:3132–54. 10.1210/jc.2009-0345 19509099

[2] Mueller A Binder H Cupisti S Hoffmann I Beckmann MW Dittrich R. Effects on the male endocrine system of long-term treatment with gonadotropin-releasing hormone agonists and estrogens in male-to-female transsexuals. Horm Metab Res. (2006) 38:183–7. 10.1055/s-2006-925198 16673210

[3] Van Cauter E Leproult R Kupfer DJ. Effects of gender and age on the levels and circadian rhythmicity of plasma cortisol. J Clin Endocrinol Metab. (1996) 81:2468–73. 10.1210/jc.81.7.2468 8675562

[4] Woods NF Carr MC Tao EY Taylor HJ Mitchell ES. Increased urinary cortisol levels during the menopausal transition. Menopause. (2006) 13:212–21. 10.1097/01.gme.0000198490.57242.2e 16645535

[5] Fonseca E Basurto L Velázquez S Zárate A. Hormone replacement therapy increases ACTH/dehydroepiandrosterone sulfate in menopause. Maturitas. (2001) 39:57–62. 10.1016/S0378-5122(01)00192-X 11451621

[6] Gudmundsson A1 Goodman B Lent S Barczi S Grace A Boyle L. Effects of estrogen replacement therapy on the circadian rhythms of serum cortisol and body temperature in postmenopausal women. Exp Gerontol. (1999) 34:809–18. 10.1016/S0531-5565(99)00044-3 10579640

[7] Komesaroff PA Esler MD Sudhir K. Estrogen supplementation attenuates glucocorticoid and catecholamine responses to mental stress in perimenopausal women. J Clin Endocrinol Metab. (1999) 84:606–10. 10.1210/jc.84.2.606 10022424

[8] Lombardo F Toselli L Grassetti D Paoli D Masciandaro P Valentini F. Hormone and genetic study in male to female transsexual patients. J Endocrinol Invest. (2013) 36:550–7. 10.3275/8813 23324476

[9] Carr BR Parker CR Jr Madden JD MacDonald PC Porter JC. Maternal plasma adrenocorticotropin and cortisol relationships throughout human pregnancy. Am J Obstet Gynecol. (1981) 139:416–22. 10.1016/0002-9378(81)90318-5 6258436

[10] Ho JT Lewis JG O'Loughlin P Bagley CJ Romero R Dekker GA. Reduced maternal corticosteroid-binding globulin and cortisol levels in pre-eclampsia and gamete recipient pregnancies. Clin Endocrinol. (2007) 66:869–77. 10.1111/j.1365-2265.2007.02826.x 17437519

[11] Genazzani AR Lemarchand-Béraud T Aubert ML Felber JP. Pattern of plasma ACTH, hGH, and cortisol during menstrual cycle. J Clin Endocrinol Metab. (1975) 41:431–7. 10.1210/jcem-41-3-431 169283

[12] Maki PM Mordecai KL Rubin LH Sundermann E Savarese A Eatough E. Menstrual cycle effects on cortisol responsivity and emotional retrieval following a psychosocial stressor. Horm Behav. (2015) 74:201–8. 10.1016/j.yhbeh.2015.06.023 26187711 PMC4876953

[13] Uhart M Chong RY Oswald L Lin PI Wand GS. Gender differences in hypothalamic-pituitary-adrenal (HPA) axis reactivity. Psychoneuroendocrinology. (2006) 31:642–52. 10.1016/j.psyneuen.2006.02.003 16616815

[14] Goel N Workman JL Lee TT Innala L Viau V. Sex differences in the HPA axis. Compr Physiol. (2014) 4:1121–55. 10.1002/cphy.c130054 24944032

[15] Chapman K Holmes M Seckl J. 11β-hydroxysteroid dehydrogenases: intracellular gate-keepers of tissue glucocorticoid action. Physiol Rev. (2013) 93:1139–206. 10.1152/physrev.00020.2012 23899562 PMC3962546

[16] Pepe GJ Burch MG Albrecht ED. Estrogen regulates 11 beta-hydroxysteroid dehydrogenase-1 and−2 localization in placental syncytiotrophoblast in the second half of primate pregnancy. Endocrinology. (2001) 142:4496–503. 10.1210/endo.142.10.8434 11564715

[17] Low SC Assaad SN Rajan V Chapman KE Edwards CR Seckl JR. Regulation of 11 beta-hydroxysteroid dehydrogenase by sex steroids *in vivo*: further evidence for the existence of a second dehydrogenase in rat kidney. J Endocrinol. (1993) 139:27–35. 10.1677/joe.0.1390027 8254291

